# Reviewer acknowledgement 2014

**DOI:** 10.1186/1750-1172-9-10

**Published:** 2014-01-30

**Authors:** Ségolène Aymé

**Affiliations:** 1 Orphanet - INSERM US14, Rare Disease Platform, 96 rue Didot, 75014 Paris, France

## Abstract

**Contributing reviewers:**

The Editors of *Orphanet Journal of Rare Diseases* would like to thank all our reviewers who have contributed to the journal in volume 8 (2013).

## 

Annemieke Aartsma-Rus

Netherlands

Marc Abramowicz

Belgium

David Adams

France

Patricia Aguilar Martinez

France

Adnan Al-Araji

United Kingdom

Maria Alessio

Italy

Veerajalandhar Allareddy

United States of America

Rando Allikmets

United States of America

Julia Almeida

Spain

Andreas Altenburg

Germany

Jeanne Amiel

France

Robert Anderson

United Kingdom

Maud Els-Marie Andersson

Norway

Corrado Angelini

Italy

Lieven Annemans

Belgium

Jean-Christophe Antoine

France

Fernando Antonanzas

Spain

Tamas Aranyi

Hungary

Eloisa Arbustini

Italy

Simone Ardern-Holmes

Australia

Benoît Arveiler

France

Patrick Aubourg

France

Karen Avraham

Israel

Olga Azevedo

Portugal

Martine Bagot

France

Kate Baker

United Kingdom

Constance Barazzone

Switzerland

Angelo Barbato

Italy

Joan Albert Barbera

Spain

Timothy Barrett

United Kingdom

Peter Barth

Netherlands

Marc Bartoli

France

Jean Bastin

France

Neil Basu

United Kingdom

Agatino Battaglia

Italy

Peter Bauer

Germany

Genevieve Baujat

France

Philip Beales

United Kingdom

Michael Beck

Germany

Juergen Behrens

Germany

Bruno Bembi

Italy

Catherine Berens

Belgium

Laura Bernardini

Italy

Francoise Bernaudin

France

Jérôme Bertherat

France

Enrico Bertini

Italy

Parayil Sankaran Bindu

India

Carl Blankart

Germany

Ken Blemings

United States of America

Olaf Bodamer

United States of America

Manfred Boehm

United States of America

Harm Bogaard

Netherlands

Amanda Bok

Belgium

Nadege Bondurand

France

Jim Bonham

United Kingdom

Jean-Paul Bonnefont

France

Luca Borradori

Switzerland

Annet Bosch

Netherlands

Laurence Bouillet

France

Sheree Boulet

United States of America

Arjan Bouman

Netherlands

Rose-Mary Boustany

Lebanon

Kym Boycott

Canada

Breidge Boyle

United Kingdom

Kaan Boztug

Austria

Bernard Brais

Canada

Mariana Brait

United States of America

Allison Brashear

United States of America

Serge Braun

France

Corstiaan Breugem

Netherlands

Riva Brik

Israel

Paz Briones

Spain

Knut Brockmann

Germany

Steven Brody

United States of America

Karen Brondum-Nielsen

Denmark

Alexander Broomfield

United Kingdom

Hakan Brorson

Sweden

Peter Bruckner

Germany

Cecilia Bucci

Italy

Daniela Buhas

Canada

Peter Burgard

Germany

Lydie Burglen

France

Elisabetta Buscarini

Italy

Gary Butler

United Kingdom

Barry Byrne

United States of America

Caglar Emre Cagliyan

Turkey

Catherine Caillaud

France

Cosima Damiana Calvano

Italy

Andrew Cant

United Kingdom

Jacqueline Capeau

France

Gabriella Captur

United Kingdom

Giuseppina Caretti

Italy

Brenna Carey

United States of America

Pedro Carmona

Spain

Carlos Casasnovas

Spain

David Cassiman

Belgium

Jean-Jacques Cassiman

Belgium

Patricia Celestino-Soper

United States of America

James Chapple

United Kingdom

Yi-Wen Chen

United States of America

Tae-Joon Cho

Korea, South

Marco Cicardi

Italy

Rolando Cimaz

Italy

Lorne Clarke

Canada

Luka Clarke

Portugal

Jill Clayton-Smith

United Kingdom

Beth Cleveland

United States of America

Alain Colige

Belgium

Giacomo Comi

Italy

Paul Coppo

France

Louise Corben

Australia

Jean-Francois Cordier

France

Yvon Cormier

Canada

Valerie Cormier-Daire

France

Denis Costello

France

André Côté

Canada

Vincent Cottin

France

Timothy Cover

United States of America

Timothy Martin Cox

United Kingdom

Helen Cross

United Kingdom

Steve Cunningham

United Kingdom

Cynthia Curry

United States of America

Lorenzo Dagna

Italy

Pu Dai

China

Erica Daina

Italy

Raymond Dalgleish

United Kingdom

Bruno Dallapiccola

Italy

Luigi Dall'Oglio

Italy

James Daniel

United Kingdom

Maria Rosaria D'Apice

Italy

Anibh Das

Germany

Hugh Dawkins

Australia

Wouter de Herder

Netherlands

Pascale De Lonlay

France

Linda De Meirleir

Belgium

Oriol de Solà-Morales

Spain

Marianne De Visser

Netherlands

François-Guillaume Debray

Belgium

Ignacio del Castillo

Spain

Christopher Denton

United Kingdom

Vincent des Portes

France

Isabelle Desguerre

France

Hervé Devilliers

France

Koen Devriendt

Belgium

Chetan Dhamne

Singapore

Maria Cristina Digilio

Italy

Charles Dinarelo

United States of America

Carlo Dionisi-Vici

Italy

Sam Doerken

Germany

Jean Donadieu

France

Lionel Donato

France

Dian Donnai

United Kingdom

Teresa Douglas

United States of America

Michael Drummond

United Kingdom

Alberto Dubrovsky

Argentina

George Dubyak

United States of America

Nicolas Dupre

Canada

Isabelle Durieu

France

Ali Dursun

Turkey

Oliver Eickelberg

Germany

Behfar Eivazi

Germany

Viktoria Eleftheriadou

United Kingdom

Harald Enzmann

Germany

Ralph Epaud

France

Boban Erovic

Austria

Kelly Evans

United States of America

Khaled Ezzedine

France

Julien Faure

France

Brigitte Fauroux

France

Conleth Feighery

Ireland

François Feillet

France

Alessandra Ferlini

Italy

Jilles Fermont

United Kingdom

Mirella Filocamo

Italy

Josef Finsterer

Austria

David FitzPatrick

United Kingdom

Arvan Fluharty

United States of America

Brent Fogel

United States of America

Thierry Forges

Luxembourg

Camille Francees

France

Brunella Franco

Italy

Marc Fransen

Belgium

Edith Frénoy

Belgium

Mike Friez

United States of America

Roseline Froissart

France

Heidi Fuller

United Kingdom

Tod Fullston

Australia

Patricia Furlong

United States of America

Lucia Gagliardi

Australia

Livia Garavelli

Italy

Angels Garcia-Cazorla

Spain

Sixto Garcia-Minaur

Spain

Abhimanyu Garg

United States of America

Gemma Gatta

Italy

Matthias Gautschi

Switzerland

Urban Geisthoff

Germany

Kerstin Gerhold

Germany

Dominique Germain

France

Santhosh Girirajan

United States of America

Cecilia Giu

Switzerland

Roberto Giugliani

Brazil

Irina Giurgea

France

Brian Godman

Sweden

Lutz Goldbeck

Germany

Victor Gordeuk

United States of America

William Gower

United States of America

John Graham

United States of America

Robert Gray

United Kingdom

David Greenberg

United States of America

John Greenlee

United States of America

Matthias Griese

Germany

Daniel Grinberg

Spain

Scott Grosse

United States of America

Stephanie Grunewald

United Kingdom

Johannes Häberle

Switzerland

Eric Hachulla

France

Smail Hadj-Rabia

France

Rosie Hague

United Kingdom

Maria Francisca Ham

Indonesia

Francoise Hamers

France

Paul Harmatz

United States of America

Dominik Hartl

Germany

Cristina Has

Germany

Takashi Hashimoto

Japan

Larisa Haupt

Australia

Masahiro Hayashi

United States of America

Adam Heathfield

United Kingdom

Yorck Hellenbroich

Germany

Raoul Hennekam

Netherlands

Angela Hernandez-Martin

Spain

Bénédicte Héron

France

Frederik Hes

Netherlands

Alex Hewitt

Australia

Wendy Heywood

United Kingdom

Helmut Hintner

Austria

Michio Hirano

United States of America

Virginie Hivert

France

Georg Hoffmann

Germany

Robert Hogan

United States of America

Daniel Hohl

Switzerland

Alexander Hoischen

Netherlands

Carla Hollak

Netherlands

Aidan Hollis

Canada

Mia Horowitz

Israel

Rita Horvath

United Kingdom

Henry Houlden

United Kingdom

Outi Hovatta

Sweden

Wills Hughes-Wilson

Sweden

Khalid Hussain

United Kingdom

Adam Hutchings

United Kingdom

Caroline Huyard

France

Susan Iannaccone

United States of America

Jan-Ichi Inokuchi

Japan

Yoshikazu Inoue

Japan

Atsushi Ishii

Japan

Anna Jansen

Belgium

Mirian Janssen

Netherlands

Viala Jérôme

France

Bing-Hua Jiang

United States of America

Dong-Kyu Jin

Korea, South

Micallef Joelle

France

Simon Johnson

United Kingdom

Roberta Joppi

Italy

Albena Jordanova

Belgium

Sushil Kabra

India

Marshall Kadin

United States of America

Emil Kakkis

United States of America

Lixin Kan

United States of America

Nobuo Kanazawa

Japan

Nesrin Karabul

Germany

Takenobu Katagiri

Japan

David Keays

Austria

Naoto Keicho

Japan

Deirdre Kelly

United Kingdom

Eitam Kerem

Israel

Babak Khoshnood

France

William Kimberling

United States of America

Taroh Kinoshita

Japan

Dimitra Kiritsi

Germany

Janbernd Kirschner

Germany

Peter Kisfali

Hungary

Sabine Klaassen

Germany

Andrea Klein

Switzerland

Wigard Kloosterman

Netherlands

Sebastian Köhler

Germany

Alfried Kohlschuetter

Germany

Zlatko Kopecki

Australia

Sailesh Kotecha

United Kingdom

Beth Kozel

United States of America

Michael Kruer

United States of America

Claudia Elisabeth Kuehni

Switzerland

Pramod Kumar

Oman

Frédéric Lador

Switzerland

Pascal Laforêt

France

Poh San Lai

Singapore

Shireen Lamande

Australia

Paul Landais

France

Judith Lasker

United States of America

Paul Lasko

Canada

Giovanna Lattanzi

Italy

Christine Lavery

United Kingdom

Martin Lavin

Australia

Martine Le Merrer

France

Helen Leonard

Australia

Gaetan Lesca

France

Nancy Leslie

United States of America

Vincenzo Leuzzi

Italy

Shuan-Pei Lin

Taiwan

Michelle Lipke

Australia

Jordi Llinares

United Kingdom

Clare Logan

United Kingdom

Jayden Logan

United Kingdom

Christian Lorson

United States of America

Qi Lu

United States of America

Maurizio Luisetti

Italy

Alan Ma

Australia

Anita MacDonald

United Kingdom

Alfred Mahr

France

Don Mahuran

Canada

Georgia Malamut

France

Fransiska Malfait

Belgium

Prashanth Mamidi

United Kingdom

Angelina Maniaol

Norway

Raffaele Manna

Italy

Hilal Maradit Kremers

United States of America

Pineda Marfa

Spain

Sandrine Marlin

France

Charles Marques

Brazil

Deborah Marsh

Australia

Angelo Valerio Marzano

Italy

Jacob Mashiah

Israel

Cibele Masotti

Switzerland

Gert Matthijs

Belgium

Maria Mavris

France

Monica Mazzucato

Italy

David McDermott

United States of America

Michael McDermott

United Kingdom

Anil Mehta

United Kingdom

Eugen Mengel

Germany

Björn Menten

Belgium

Fredrik Mertens

Sweden

Albee Messing

United States of America

Jacques Michaud

Canada

Helen Michelakakis

Greece

Rado Mihai

United Kingdom

Jose Millan

Spain

Pramod Mistry

United States of America

Hiroshi Mizushima

Japan

Gabriela Moeslein

Germany

Aladdin Mohammad

Sweden

Klaus Mohnike

Ghana

Nicole Monnier

France

Ute Moog

Germany

María-Josefa Morán-Jiménez

Spain

Eva Morava

United States of America

Bruce Morland

United Kingdom

Deborah Morris-Rosendahl

United Kingdom

Despina Moshous

France

Joseph Muenzer

United States of America

Jean Muller

France

Stefan Mundlos

Germany

Francisco Muñoz-Beamud

Spain

Simon Murch

United Kingdom

Elaine Murphy

United Kingdom

Joseph Murray

United States of America

Dedee Murrell

Australia

Satoshi Narumi

Japan

Gabor Nemeth

Hungary

Anne Nepo

United States of America

William Newman

United Kingdom

Denise Ney

United States of America

Garth Nicholson

Australia

Elena Nicod

United Kingdom

Ichizo Nishino

Japan

Dean Nizetic

United Kingdom

Eduardo Nobile-Orazio

Italy

Markus Nöthen

Germany

Giuseppe Novelli

Italy

Mark O'Driscoll

United Kingdom

Vinzenz Oji

Germany

Torayuki Okuyama

Japan

Nicolas Ortonne

France

Daniel Ory

United States of America

Shahrzad Ossareh

Iran

Chris Ottolenghi

France

Laurie Ozelius

United States of America

Richard Paisey

United Kingdom

Davide Pareyson

Italy

Rossella Parini

Italy

Eduard Paschke

Austria

Gregory Pastores

United States of America

Marc Patterson

United States of America

Kyle Peake

Canada

Marieke Peetsold

Netherlands

Alan Percy

United States of America

Gabriela Petrof

United Kingdom

Flora Peyvandi

Italy

Eline Picavet

Belgium

Robert Pignolo

United States of America

Wim Pinxten

Belgium

Vinciane Pirard

Belgium

Francesco Pisani

Italy

Violaine Plante Bordeneuve

France

Barbara Plecko

Switzerland

Miguel Pocovi

Spain

Petr Pohunek

Czech Republic

Venerino Poletti

Italy

Robert Pomponio

United States of America

Raphael Porcher

France

Berardino Porfirio

Italy

Forbes Porter

United States of America

Carmen Priolo

United States of America

Ewa Pronicka

Poland

Catherine Prost-Squarcioni

France

Alexey Pshezhetsky

Canada

Maria Puiu

Romania

Stefan Pulst

United States of America

Waseem Qasim

United Kingdom

Pierre Quartier

France

Elzbieta Radzikowska

Poland

Shamima Rahman

United Kingdom

Julian Raiman

Canada

Philippe Reix

France

Arnold Reuser

Netherlands

Vincent Riccardi

United States of America

Lisa Riley

Australia

Mara Riminucci

Italy

Carlo Rivolta

Switzerland

Leema Robert

United Kingdom

Rhys Roberts

United Kingdom

Hugo Rocha

Portugal

Júlio Rocha

Portugal

Natasa Rojnic Putarek

Croatia

Jill Rosenfeld

United States of America

Philip Rosenthal

United States of America

Andrea Rossi

Italy

Johannes Ruige

Belgium

Heiko Runz

Germany

Nicolino Ruperto

Italy

David Saadoun

France

Denise Sabatino

United States of America

Carlo Sabbà

Italy

Inderneel Sahai

United States of America

Lynn Sakai

United States of America

Ettore Salsano

Italy

Leonardo Salviati

Italy

Robert Sandhaus

United States of America

Luca Sangiorgi

Italy

Damien Sanlaville

France

Gijs Santen

Netherlands

Carol Saunders

United States of America

Maurizio Scarpa

Italy

Chayim Schell-Apacik

Germany

Angelo Schenone

Italy

Arrigo Schieppati

Italy

Matthias Schmuth

Austria

Ludger Schöls

Germany

Elizabeth Schorry

United States of America

Benedikt Schoser

Germany

Jacqueline Schoumans

Switzerland

Richard Schreiber

Canada

Andreas Schulze

Canada

Alfredo Scillitani

Italy

Marie Scully

United Kingdom

Frederic Sedel

France

Petra Seemann

Germany

Bruno Sepodes

Portugal

Andreas Serra

Switzerland

Trevor Shepherd

Canada

Eamonn Sheridan

United Kingdom

Juhong Shi

China

Haruo Shintaku

Japan

Yehuda Shoenfeld

Israel

Cheryl Shoubridge

Australia

Elena Sieni

Italy

Eduardo Silva

Portugal

Helga Silva

Brazil

Steven Simoens

Belgium

Alessandro Simonati

Italy

Natalie Sims

Australia

Rani Singh

United States of America

Siddharth Singh

United States of America

Cassian Sitaru

Germany

Bert Smeets

Netherlands

Mike Soucie

Uzbekistan

Miles Stanford

United Kingdom

Matthew Steensma

United States of America

Colin Steward

United Kingdom

Sylvia Stockler

Canada

Jan Stolk

Netherlands

Björn Stollenwerk

Germany

Arnold Strauss

United States of America

Robert Strohal

Austria

Oksana Suchowersky

Canada

Mohnish Suri

United Kingdom

Jolanta Sykut-Cegielska

Poland

Matthis Synofzik

Germany

Atsuhito Takeda

Japan

Chantal Tallaksen

Norway

Clara Tang

Hong Kong

Marc Tardieu

France

Simon Taylor

France

Samia Temtamy

Egypt

Sharon Terry

United States of America

Christoph Thiemermann

United Kingdom

Cynthia Tifft

United States of America

Vallo Tillmann

Estonia

Dagmar Timmann

Germany

Vincent Timmerman

Belgium

Sigrid Tinschert

Austria

Valeria Tiranti

Italy

Maarten Titulaer

Netherlands

Simrandeep Tiwana

Canada

Roser Torra

Spain

Antonio Torrelo

Spain

Alfredo Torres

United States of America

Antonio Toscano

Italy

Isabelle Touitou

France

Valerie Touitou

France

Mondher Toumi

France

Juan Tovar

Spain

Elias Traboulsi

United States of America

Lisbeth Tranebjaerg

Denmark

Eileen Treacy

Ireland

Friedrich Trefz

Germany

James Triffitt

United Kingdom

Barbara Triggs-Raine

Canada

Michael Tropak

Canada

Lennart Truedsson

Sweden

Edward Tuddenham

United Kingdom

Sylvie Tuffery-Giraud

France

Peter Turnpenny

United Kingdom

Ifigeneia Tzannou

United States of America

Argyris Tzouvelekis

Greece

Bjarne Udd

Finland

Elena Urcelay

Spain

Graziella Uziel

Italy

Augusto Vaglio

Italy

Filippo Vairo

Brazil

Vassili Valayannopoulos

France

Enza Maria Valente

Italy

Dominique Valeyre

France

Laurence Valeyrie-Allanore

France

Ludo Van den Bosch

Belgium

Ans van der Ploeg

Netherlands

Gert Van Goethem

Belgium

GertJan van Ommen

Netherlands

Sonja van Weely

Netherlands

Anthony Vandersteen

United Kingdom

Marie Vanier

France

Timothy Vece

United States of America

Ashok Vellodi

United Kingdom

Charles Venditti

United States of America

Tiziana Venesio

Italy

Alain Verloes

France

Lluïsa Vilageliu

Spain

Maria-Antonia Vilaseca

Spain

Ricardo Villa-Bellosta

Spain

Josip Vincelj

Croatia

John Vincent

Canada

Hannes Vogel

United States of America

Annick Vogels

Belgium

Klaus Wagner

Austria

Thomas Wagner

Germany

Stephen Waldek

United Kingdom

John Walter

United Kingdom

Corry Weemaes

Netherlands

Frank Weidemann

Germany

Sheila Weitzman

Canada

David Wenger

United States of America

Michael West

Canada

Kerstin Westermark

Sweden

Patrick Whelan

United States of America

Gabrielle Wheway

United Kingdom

Dagmar Wieczorek

Germany

Frits Wijburg

Netherlands

Bridget Wilcken

Australia

Andrew Wilkie

United Kingdom

Michael Wilschanski

Israel

Ingegerd Witt Engerström

Sweden

Malgorzata Wiweger

Poland

Chen-Chi Wu

Taiwan

Kaleb Yohay

United States of America

Toshifuymi Yokota

Canada

Pierre Youinou

France

Peter Young

Germany

Paul Yu

United States of America

Bernhard Zabel

Germany

Francesco Zambon

Italy

Giovanna Zambruno

Italy

Christina Zeitz

France

Ramzi Zemni

Tunisia

Massimo Zeviani

Italy

Xue Zhang

China

Zhigang Zhang

China

Jia Wei Zheng

China

Manfred Zierhut

Germany

Vlasta Zmazek

Croatia

Thomas Zoller

Germany

Christos Zouboulis

Germany

Gabor Zsurka

Germany

Stephan Zuchner

United States of America

Christiane Zweier

Germany

